# Learning Activity as a Means of Developing Theoretical Thinking Capacities

**DOI:** 10.3389/fpsyg.2020.603753

**Published:** 2020-12-14

**Authors:** Cleber Barbosa da Silva Clarindo, Stella Miller, Érika Christina Kohle

**Affiliations:** Postgraduate Program in Education, Faculty of Philosophy and Science, Universidade Estadual Paulista (UNESP), São Paulo, Brazil

**Keywords:** capacities of theoretical thinking, analysis, reflection, mental planning, organization of learning activity

## Abstract

The main purpose of this article is to discuss the development of capacities linked to theoretical thinking during the formation process of Learning Activity in students of the early years of elementary school. It considers some elements which could form the basis for thinking about teaching activity as a means of conducting students toward that development. It starts from the hypothesis that the development of the capacities of analysis, reflection, and mental planning depends, essentially, on the systematic organization of the principles and ways of action of the students on the theoretical contents in their learning process. The hypothesis further states that Learning Activity is the most appropriate way to organize pedagogical work for the development of methods of action that would enable students to achieve new higher psychological formations and develop new ways of thinking and acting in the learning environment. The theoretical study was carried out through locating, gathering, analyzing, synthesizing, and reframing the theorical ideas contained in publications, texts, articles, and books by authors of the Cultural-Historical Theory. The study places emphasis on Learning Activity as an adequate way to develop psychic neoformations in school-age children, since their organization, based on the solution of learning tasks through learning actions, is the source of the development of analysis, reflection, and mental planning skills, as constituent parts of theoretical thinking in school-age children.

## Introduction

This article discusses the development of capacities linked to theoretical thinking during the formation process of Learning Activity in students of the early years of elementary school. It considers some elements which could form the basis for thinking about the teaching activity as a means of guiding students toward that development. The results presented here come from research that was finished in 2020, entitled *Learning Activity as a means for the development of theoretical thinking capacities* (Author, 2020, our translation), which aimed to highlight the relevance of Learning Activity for the development of capacities associated with theoretical thinking - analysis, reflection, and mental planning - as neoformations to be developed at school age.

We started from the assumption that, from a very young age, children, in any activity they perform, always learn something new in the relationship they establish with other social subjects and with the cultural objects with which they interact. They have the ability to discover and find out, in different ways, the news of the world, in such a surprising process that, in most cases, adults do not realize how children have appropriated knowledge, nor do they notice how “under their orientation, the children begin to assign meaning to the acquired knowledge” (Elkonin, 2019, p. 159). When playing with modeling clay, for example, the child is not interested in the object itself (Is the clay soft or hard?), but in what that object allows him to do, and experiences “a process of cognition and learn[s] to interact with the acquired information” (Elkonin, 2019, p. 159) (The child knows the plasticity of the object, learn that it can be modeled, etc.).

However, it is not only in relation to objects that children learn; their needs and interests expand to the knowledge of the facts and the phenomena that happen in it. In their surrounding relationships, seeking to know and understand what is going on around them, young children ask many questions to the adults with whom they live. Questions such as: Where does rain come from? How did that baby get in your belly? Do ants sleep? Why is the sky blue? They are the expression of the children’s curiosity. However, adults’ answers to their questions are often unsatisfactory for children, and they often must make up their own hypotheses and theories to satisfy their thirst for knowledge.

Nowadays, from early childhood, children use sources other than the adults in their lives in the process of appropriating information, such as television programs, literary papers, but mainly the Internet via computers, cell phones, and tablets, which are tools arising from technological changes throughout human history. These sources, many times replacing adults as a means of searching for information, can also lead to various situations in which the child ends up having better knowledge of some phenomena than the adults themselves.

Cognitive interests are often present in children before they get into the world of systematized education. So when they come to school they already have a broad view of the world acquired from their own life experiences, through which they appropriate the knowledge about the phenomena and events of everyday life. Due to its sporadic and non-systematized nature, this form of knowledge appropriation leads to the development of so-called spontaneous concepts, in opposition to scientific concepts.

We say that scientific concepts are developed in children in a different way than spontaneous ones and by other means. It seems to me that the very fact that school learning - the child studies for the first time a system of some scientific knowledge - is so sharply distinguished from the conditions in which his first concepts arise, allows us to suppose that the development path to scientific concepts is another one. But it is clear that one should not overestimate the differences between the ways in which spontaneous and scientific concepts arise. And you can’t do it from two points of view. Because learning doesn’t just start at school age. When the child asks “Why?” and the adult answers, when she hears stories told by an adult or other children, she is actually learning (Vigotski, 2010, p. 524–525).

Although going to school does not stop the process of appropriating knowledge through the experiences of everyday life, it introduces children to a new activity that marks the way in which they learn and develop their lives in this period of learning: Learning Activity, a new way of relating to knowledge that can change children’s ways of dealing with reality.

Learning Activity has both changing features and content characteristics: it is an activity that allows school-age children to appropriate contents that are based on the most advanced forms of knowledge that humanity has produced - scientific knowledge. In the process of assimilating this content, children perform learning tasks that require the use of analysis, reflection, and mental planning, thereby developing a complex form of thinking: theoretical thinking. Because of its transformative potential, the Learning Activity, and the pedagogical organization that derives from it, should assume the fundamental role of overcoming the traditional forms of education that reproduce and are means of maintaining the *status quo*. Thus, this new pedagogical organization would form creative subjects, capable of looking critically at reality, of understanding the facts, phenomena, and events of their environment, and who are also capable of acting in it in order to transform it.

Given this changing feature of Learning Activity, it is essential to think of it as a means for organizing an emancipatory educational practice for school-age children. Learning Activity aims to develop in children the capacity to allow them to understand reality, giving them the capacity for reflection, analysis, and mental planning, which are essential elements that make up their theoretical consciousness, and which concretize their physical relationship and theoretical relationship with the world.

## Methodology

The research that resulted in the data, part of which is presented in this article, is theoretical research. It is based on data collection in papers related to the topic discussed here, on the gathering of data generated from a bibliographic search, on the analysis of these data, and, finally, on the synthesis and reframing of the theorizations contained in the different consulted publications. The research special focus was on articles from scientific journals and books by authors from the Cultural-Historical Theory that dealt with Learning Activity as a way in which the psychic neoformations of school-aged children are developed.

This type of research aims to “broaden generalizations, define broader laws, structure theoretical systems and models, relate and link hypotheses in a more unitary view of the universe, and generate new hypotheses due to logical deduction” (Oliveira, 1997, p. 123, our translation). For its fulfillment, bibliographic research was made, which constitutes a fundamental resource, since it “aims to know the different forms of scientific contribution that have been made on a certain subject or phenomenon” (Oliveira, 1997, p. 119, our translation).

The studies carried out before this research highlighted the need to deepen the knowledge about how human capacities are developed through Learning Activity and how they lead to the total formation of theoretical thinking, that is, to the unit that constitutes theoretical-reflective thinking. So, we looked for it.

The research was, therefore, designed with the objective of understanding the way in which the neoformations of the psyche of school-age children are organized. That is to say, how the capacities of analysis, reflection, and mental planning, related to theoretical thinking are organized in an interrelational system that is constituted in the process of development of Learning Activity.

Advances in research on Learning Activity are fundamental both to overcome, in the real practice of schools, the conceptions of non-emancipatory education, which use educational processes to maintain the *status quo*, and to build up and implement developmental pedagogical practices. These kinds of practices cause qualitative changes in students’ psyche, since, through the practical and revolutionary transformation of the teaching-learning processes, it is possible to build an education that has a truly transformative role.

## Development

School education and Learning Activity are not isolated from activities in other areas of life. This fact leads to a certain multiplicity of interests between Learning Activity and other activities of children’s daily life. Thus, there are cases in which, even before starting school, the child already has favorite activities, which he performs as leisure or hobby. At the beginning of schooling this fact can lead children to think that school is an obligation that can hinder their favorite activity. In this context, Learning Activity will not, automatically or immediately, replace children’s main activity upon their arrival in the school environment.

However, since the first moments of their admission to this institution, children have already experienced the importance of their actions as students. All the essential processes in their development are directly related to their new role, both within the family and within the school structure, as their ways of acting are directly connected and supported by this new social position. The feelings that emerge in such a social position make the children assign a personal purpose to the activity that, at that moment, is not yet configured for them as a Learning Activity; this activity must be formed as such for the learner to feel the necessity of his theoretical capabilities’ development.

For this reason, the social role of a student, which motivates children at the beginning of their school life, does not last long. This is because at some point it will lose its motivating importance, and it will be replaced by another tasks. Depending on how pedagogical practice is organized, it will not create or motivate Learning Activity. The motivation for Learning Activity is, therefore, of fundamental importance and is directly linked to the issue of the child’s needs. Indeed,

Motivation cannot […] be understood outside the scope of need, nor outside the scope of activity, since they maintain a close link with the overall situation experienced by the individual, both from an organic point of view (biological sense) and from the point of view of personality (personal sense), or from the point of view of the meaning (social sense) of the activity. Yet, it is necessary to affirm that these forms of meaning are integrated in the psychological orientation, merging in a particular way of objectifying the actions.

Motivation is full of meaning and orientation; it represents the complexity of the person’s active character in relation to his activity, and is directed to the content of the need, that is, to its object (Marino Filho, 2011, p 127–128, our translation, emphasis in the original).

If the purpose of the child’s need is primarily directed toward goals, such as seeking approval from parents and teachers, receiving awards for the good results of their learning, or being valued for their role as a student, the reasons that derive from it are characterized as private and “act for a short time and under direct circumstances (for example, the teacher’s attitude)” (Leontiev, 1960, p. 349). For this reason, it is possible to find, at the end of the first year of study, children who no longer have the same motivation to perform tasks related to the social role of students.

According to Davídov (2019), the weakening of the motivating role of social position has two causes. The first one is related to the consolidation of the student’s own social position: as it has already been achieved by the child, by coming to school and having the consequent recognition of his role linked to that position, no great effort is required to maintain that status before society. The second cause refers to the fact that the motivation that derives from social position has its origin in external factors of the learning process. In other words, there is no direct relationship between the motivational function of social position and the way in which students appropriate the contents of school subjects in the classroom: social position must not be confused with what is necessary to learn and study, since motivation to study associated with the social position is replaced by reasons based on competition and obligation to perform school tasks, which are external to the subject of learning. The consequence of this external origin of the motives for studying is the disagreement between the social position and the contents of the activities in which the student is involved in the classroom, leading students to lose, often in the first years, their interest in the school activity.

In order to guide children to genuine Learning Activity, whose content of study corresponds to the substance of their needs, it is necessary to develop in them general and broad reasons, which “are more constant, operate for a long time, and do not depend on casual situations” (Leontiev, 1960, p. 349, our translation). However, it is necessary to consider that “[.] the reasons may have a distinct relationship with the possibility of carrying out the activity that originates them. In order for a reason to really cause an activity, there must be conditions that allow the subject to project the corresponding aim and act to achieve it. Only in this case is the reason effective” (Leontiev, 1960, p. 348, our translation). Therefore, it is essential to create needs and reasons for the development of Learning Activity.

### Creating the Reason for Learning Activity

All forms of human activity begin with the presence of a need realized by the subject, as it is configured as “a mental state that supplies the precondition for activity” (Markova, 1986, p. 15), and that is no different for Learning Activity. Despite this condition, the need in itself does not guarantee the advent of reasons for a school child to enter into activity. “But in the absence of a perceived need, the child’s activity is never aroused; no motive emerges, and he is in no way ready to start setting goals for himself” (Markova, 1986, p. 15).

From this point of view, the development of Learning Activity can only take place when the reasons that correspond to the content of the educational process and that direct this activity to satisfy a learner’s need are formed. Only then does the student become interested in appropriating the knowledge inherent in that activity. The reason is, therefore,

[…] the directed [or orientational] nature of activity with respect to a given object or subject, and the inner mental state that has direct links with the objective characteristics of the item toward which the activity is directed. While the perceived need indicates a readiness to undertake activity, this activity is more effective in the presence of motive (Markova, 1986, p. 17).

The most complex activities, such as Learning Activity, relate to more than one reason simultaneously. In addition to the more general and broad reason that directs human action to the central focus of the activity, there are particular and restricted reasons, external to the central activity that, with the general and broad reasons, form a system of reasons each with its specific role: the broader and more general reason gives a certain meaning to the activity, and the others drive the subject to immediate action (Leontiev, 1960).

In [formal] teaching and learning, motive means the directed quality of pupil activity relative to individual facets of the learning process. In plain fact, the pupil’s drive to master knowledge, to get good grades, to win parental praise, and to form desired relationships with his peers are all part of this. In other words, learning behavior is always stimulated by any number of motives (Markova, 1986, p. 17).

When the learning reasons are directed to the appropriation, by school children, of the methods aimed at scientific knowledge and the knowledge that is originated from it, so that they know how to plan, analyze, and reflect about what steps must be taken in the constant process of knowing reality, the activity has a vital meaning for them and keeps them attentive and dedicated to their study. For this reason, developing Learning Activity in such a way that this activity is the main source of attributing personal sense to the student’s life within the school environment and outside of it – a sense that expresses what he considers to be of great relevance to his life - is among the most important objectives of children’s education in the early years of elementary school.

Seriously considering the motivational sphere in the organization of Learning Activity is, therefore, a core issue for the effective conduct of the teaching and learning processes in schools. It is evident that our educational system needs to pay attention to the reasons that lead the child to study or not, as well as to analyze the teaching-learning process from the point of view of motivation for learning. As Elkonin, 2019, p. 161, our translation) points out, “Learning problems can only be solved in the conditions of motive formation for Learning Activity,” since they direct the people’s actions toward objectives to be achieved within certain conditions for carrying out this activity, that is to say, give the direction for the fulfillment of the learning tasks. The learning task, as the main component of Learning Activity, requires the child to carry out mental processes that go from the initial analysis of the learning object to the construction of a substantial abstraction and generalization to discover the general relationship that organizes it, passing through the deduction of the particular relations of that object and the elaboration of a synthesis that expresses the totality of the object, until complete mastery of a general procedure of studying the object is gained. This movement realizes “a certain microcycle of ascension, from the abstract to the concrete, as a way of assimilating theoretical knowledge” (Davídov, 1988, p. 179, our translation) which culminates in the qualitative transformation of the mental processes of the learners.

From this point of view, Learning Activity differs from other activities that have a material object as a final product, such as a woodworker, who works with the manufacturing of chairs, or a child who plays at building cakes in the sand, or another child who draws a car or a boat on a sheet of paper. All of these activities result in the production of a material object, because, during these activities, people introduce changes in a certain element to obtain the final and material product of their actions.

On the other hand, it cannot be denied that during work activities, playing time, artistic actions, or any other activities, transformations occur in the capacities of the subject of the activity. However, these transformations are not the main product of the process. At work, for example, it is not intended for the final content of the subject’s actions to be a transformation in his personality. At work, it is clear that it is a secondary concern whether or not changes occur in the people who are part of the production process; what is essential is the final product and its qualities as a material object that has an established function within the production process. As Davídov (2019) states,

The assimilation of knowledge, skills, and habits comes from communicating with parents and peers, playing and reading books. The man gains great experience in the work process. What is the peculiarity of the assimilation that operates in Learning Activity? First of all, it is necessary to remember that the conditions for Learning Activity to be carried out with amplitude are created only at school, where the fundamentals of science are learned and the scientific conception of the world is still being formed. The content of Learning Activity has a distinct peculiarity: its basic part consists of *scientific concepts, laws of science, and generalized ways* of solving practical problems based on these laws and concepts (Davídov, 2019, p. 182, our translation, emphasis in the original).

In this activity, students use and apply theoretical concepts through the guidance of the teacher, but they do not transform the general system of these concepts. The fact that the child works with them does not result in a total change of science, on the contrary, the transformations that students experience in Learning Activity happen in themselves, when appropriating scientific knowledge and developing skills and abilities. For this reason, we can say that Learning Activity is an activity of self-transformation of the student, and that the essential result will always be the constant development of the personality of the individual who is in activity. This characteristic differentiates Learning Activity from other activities.

In other types of activity, assimilation appears as an additional product. For example, during play time, the child has to fulfill the role he has assumed in the best possible way. In assuming it, he assimilates only the rules of conduct accompanied by the satisfaction of the fundamental desire. In turn, the main objective of the work is the production of objects. The enrichment of attitudes only appears as an important result, but it is an accessory to the work. In Learning Activity, the fundamental and main objective is the assimilation of scientific knowledge and corresponding attitudes (Davídov, 2019, p. 182, our translation).

However, Learning Activity also produces material results. In the written production process, for example, the results are the written statements of the most diverse discursive genres; when solving mathematical questions, material production is the solution given to arithmetic and algebraic problems. However, this material production should be seen as the process of identifying and evaluating the actions that are taken by the students regarding the qualitative changes that they went through during the execution of Learning Activity. In other words, “The results are evaluated from the point of view of the changes caused to the student and not from the point of view of their social utility” (Elkonin, 2019, p. 162, our translation) as the results of the work activity are evaluated.

Even dealing with the same field of educational activity, there were approaches that presented divergences in the conception of the teaching-learning process. Teaching methods based on traditional formal logic, for example, unlike the dynamic proposed for Learning Activity, focus their attention on the amount of knowledge that students were able to acquire after a period of study. The results of the evaluations show more what was memorized and remembered by the student than the qualitative changes that occurred in his psyche, expressed in terms of new capacities developed by him, thus showing his character of reproduction and maintenance of the *status quo.*

Regarding the self-transforming character of Learning Activity, and considering that its objective is the appropriation of the principles that result in the acquisition of the scientific knowledge, the appropriation of adequate modes of motivation that produce a positive attitude in the subject in relation to the study and its consequent active adherence to this activity are necessary.

### The Methods of Action in Learning Activity

Learning Activity is the guiding activity of initial school-age children, since qualitative leaps in the development of children in this stage take place through this activity. In the set of human beings’ forms of activity, Learning Activity is the one that symbolizes the main way of acting for children who are starting a systematized education; through it, children appropriate the basic elements of the most elaborate forms of knowledge of the objective reality that, historically, were built by humanity, such as philosophy, science, and the arts, which allow humans to understand the world beyond the limits of everyday life.

Upon entering the world of systematized knowledge, children will totally transform their ways of action into reality. Initially they change their position in social relations, which takes place both at school and in their home environment, due to radical changes in the content and character of their activities. In the Learning Activity, children will have, for the first time, the awareness that their abilities have a creative power, and also that their actions have the power to transform reality. In addition, for the first time, they will have autonomy to control these ways of action, with their own development as the main goal.

As a result, Learning Activity is, for children in their first years of elementary school, the qualitative form of development for their personality, a process that happens due to the appropriation of the most elaborated cultural assets in the historical development of humanity. Scientific knowledge corresponds to those more developed cultural forms that school-age children should appropriate. They are characterized by a double function: they constitute the content of Learning Activity and, at the same time, they are the motivating elements for the student to form the need to appropriate the capacities of theoretical thinking. And this process is always active: there must be an action made by the subject on the learning object in a situation of interaction with other subjects involved in it. In other words,

In educational process, from schooling point of view, the object of learning appears to learning subject as an objectification historically elaborated by other subjects who preceded him/her. By means of an activity intentionally directed to the object learning, establishing relationships with his peers and with the teacher, the student appropriates that object and thus he/she becomes able to objectify himself/herself in new products of his/her activity. (Miller, 2019, p. 73, our translation).

In Learning Activity, the process of appropriation of scientific knowledge occurs through the solution, by the students, of the learning tasks, since it is in the learning task that the child is allowed to discover the conditions of the origin of scientific knowledge. It stimulates the search for what is not known and the consequent assimilation of the new knowledge and methods of action, which will give meaning to the activity overall, since the task is the unity between the objective of the action and the conditions for its fulfillment.

The learning task requires the students to take types of procedures that begin with the objective analysis of the material in order to discover the general relationships that present themselves in its various manifestations, in a process of building essential abstractions and generalizations of knowledge. The second procedure is in deduction, through essential abstractions and generalizations, of the particular relationships of the studied material, leading to the synthesis of the object, and the consequent construction of a cell that expresses the object in its essential integrality. And, finally, in the learning task, the mastery of general procedures needed to approach the mental object in an analytical-synthetic process is established.

This learning task movement does not appear when the teaching-learning process is guided by methods based on traditional formal logic, mentioned above, as they deal with the transmission of consolidated knowledge, for which students are presented objectively by means of verbal abstractions, developed successively, and linked to sensorial images that are determined and visibly accurate about the knowledge presented. This type of approach induces students to develop the empirical classifying peculiarities in their thinking (Davydov, 1981), in contrast to the theoretical thinking developed by Learning Activity.

In other words, this logical-formal approach to the content characterizes the “empirical theory of abstraction, generalization, and the formation of concepts” whereby there is no “qualitative difference between daily and scientific concepts. They are distinguished only by the volume and systematized character of the knowledge equally accessible in principle to everyday and scientific experiment” (Davídov, 1986, p. 236, our translation). The awareness of knowledge, in this perspective, is therefore limited, since the concept acquired by the student is reduced to the appropriation of external aspects of knowledge, linked merely to practice and fixed by the word. Therefore, there is no development of more complex cognitive abilities and creative independence in students, as proposed by Learning Activity.

Differently, the materialization of the learning task happens through learning actions that, in the process of the appropriation of scientific knowledge, are internalized in theoretical thinking according to the principle of thinking as action: the learning actions are the ones that move the act of thinking and constitute the capacities of theoretical thinking in learners.

In the sequence, the specificities of the elements that constitute the three capacities of theoretical thinking will be discussed.

#### The Capacity for Analysis

Learning Activity, organized on the basis of scientific knowledge, which is intentionally directed to the development of the student’s theoretical and productive thinking, has as its structural unit the learning task (learning actions directed to specific objectives that are carried out in accordance with certain conditions).

In the development of the learning task, the first action that is presented to the student is to carry out an analysis, still in the object-sensory area, of the task content. In the course of this initial action, “the transformation of the learning task data takes place in order to reveal a certain universal relationship of the given object, which must be reflected in the corresponding theoretical concept” (Davídov, 1988, p. 182, our translation).

The appropriation of scientific knowledge and the development of skills and abilities linked to the formation of theoretical-conceptual thinking are at stake in this process. This appropriation of knowledge “presupposes the study, by the children, of the original conditions of the corresponding concepts, which, in turn, forms in them the systems of mental actions that allow them to operate properly with these concepts” (Davídov and Márkova, 1987, p. 177, our translation). And this is only possible through the theoretical analysis of the learning task content, to be solved as part of a Learning Activity inserted in a problem-solving situation.

When we talk about theoretical analysis of the content of a learning task within a problem-solving situation, we are dealing with a concept whose comprehension demands the distinction between what is considered its objective and logical content and what its subjective and psychological function is. From an objective and logical point of view, the theoretical analysis of the content can be defined as

[…] a means of distinguishing the genetically original relationship underlying a particular object system — in other words, as a starting point for the movement (ascent) of theoretical knowledge from the abstract to the concrete. On the subjective and psychological side, as it appears in a problem-solving situation, analysis functions as the search for the problem’s main relationship and the principle of its solution (Magkaev, 2018, p. 304).

The search for this main and universal relationship as a subjective and psychological function of theoretical analysis, in accordance with its objective and logical content that establishes the genetically original relationship of a system of objects, configures the initial stage of the concept formation process that is present in the learning object. Through theoretical analysis, the subject reaches a genetically initial abstraction that expresses the essence of this object, defined as “the internal connection that, as a single source, as a genetic basis, determines all the other particularities of the whole,” configuring the “objective links, which in their dismemberment and manifestation ensure the unity of the aspects of the whole, that is, they give the object a concrete character” (Davídov, 1988, p. 147).

At this point in the development of Learning Activity, the teacher directs the student’s learning task toward the theoretical analysis of its content, taking into account the conditions and means that determine it, considering that the theoretical analysis is fully satisfactory only if the subject is able to analyze the range of circumstances of the task and distinguish what is essential from what is accessory, thereby avoiding, through the general procedure of solving the learning task, the consideration of accessory circumstances that are not essential to their solution. In other words, the teacher starts the actions related to the learning task knowing that analytic action reveals, based on an aspect, characteristic, or quality, the essential relation of the problem, through continuous changes in the forms of exploration of the provided material conditions - in the identification of what is given and known, and in the differentiation of what is unknown and hidden.

In this process movement, the student develops the capacity for theoretical analysis that manifests itself in the seeking, in the identification, and in the appropriation of genetic and original relationships of a set of particular objects. The students also appropriate their own role in this process, which is that of identifying the essential relationships, or the principles that are implicit in the objective resolution of the problem, in an inseparable way from the conditions, means, and purposes of the problem-solving task.

In order to think about and elaborate on the total concept of theoretical analysis, it is fundamental to understand that the seeking and construction, by the subject, of the problem-solving principle involves the relationship and connection of all components of the given situation in a mental whole, in a conceptual image of the problem situation. In other words, the conceptual construction of the analytical action must be based on understanding the set of interconnections that constitutes a conceptual image of the problem situation.

In this process, the possibilities of connections and relationships established and their unique quality depend directly on the particular content of the problem and on the ways in which it presents itself. However, whatever the particularities of the problem situation may be, the constitution of a conceptual image will always require total understanding of it, which, in other words, means the connection of all relationships and links that present them in the process of solving the problem.

When thinking about the objective questions of analytical capacity, we can establish that, as a result of the seeking, experimentation, and transforming activity of the subjects, the integral conceptual image of the problem-situation is constituted, leading to the revelation of the essential relation of the problem. From a subjective and psychological point of view, this process results in the subject’s conception of an ideal or mental form related to the possibility of action on the object of knowledge as on its objectives that are represented in the form of a hypothesis or a set of possibilities to act.

To clearly define the understanding of the theoretical analysis content, it is necessary to visibly identify the contents of the following movements: the first refers to the necessary comprehension of the genetic origin of this capacity in the process of developing a system of knowledge objects in its logical category; the second is concerned with the search for the essential relation of the constructed object content; and the third is about seeking the essential principle that can solve the problem, that is the creation of generalizations to solve all problems of the same class (Magkaev, 2018). These movements related to theoretical analysis characterize the actions of reflective-theoretical thinking aimed at understanding reality. They constitute a path for the comprehension of the totality in its real form, in movement, through the abstract to the concrete conceived as a synthesis of multiple determinations.

A relevant aspect of the analytical action constitution is its relation to the synthesis process. Throughout the trajectory undertaken to solve a problem, the analysis operation and its opposite operation, the synthesis, are fundamental: the analysis decomposes the problem into its elements, and the synthesis brings them back together in order to solve them, obtaining as a result the view of the object as a totality. This object arises, then, with a qualitatively different form from the object seen in the starting point for the resolution of the learning task. As a consequence, in this process of analyzing and systematizing, the thought goes from a vague initial representation of the object to the formation of the concept, the general idea that analytically constitutes itself as essential elements and, systematically, presents the essential relationships of the whole (Petrovsky, 1980).

There lies the relevance of developing the capacity for analysis and synthesis in the process of forming Learning Activity, since “*Analysis and synthesis* are the fundamental rational operations [.] [that] occupy a special place in all mental operations. Every thought is a cerebral analytical-synthetic function and consists of different degrees of analysis and synthesis” (Gurevich, 1960, p. 236, our translation, emphasis in the original).

These two operations must always converge as a whole in the process of real appropriation of scientific knowledge. In essence, they remain inseparable and develop with an intimate dialectical relationship, but, in certain situations of the knowledge search procedure, they can predominate and come to the forefront of the mental process development. The predominance of analysis or synthesis in a given period of the mental process can be conditioned by the classes of elements or phenomena. For example, if the initial data of the phenomena and elements of the problem are unclear, then, in the first stages of mental action, analysis predominates. In turn, if at the beginning of the mental process all the data are already clear enough, the thought will follow promptly and the path will be preferably that of synthesis.

Although *analysis and synthesis* are two opposing operations, *they are linked inseparably*. When we read, we separate different phrases, words, and letters from the text and, at the same time, we connect them with each other: the letters we put together in words, the words in sentences, the sentences in one or another part of the text. When an event is reported, some separate episodes mentally disintegrate; but at the same time, their relationship with each other, their dependence, is marked. The same is true in all thought activities (Gurevich, 1960, p. 237, our translation, italics in the original).

For the execution of the mental analysis, which aims to divide the whole into parts, it is necessary that, in the analysis process, the subject perceives what is fundamental and what is not fundamental and separates what is fundamental from the whole. In the same way, mental synthesis can be done with more precision if the subject perceives the whole that has been dismembered and tries to reconstruct it mentally by bringing together the isolated parts. In any case, when the reconstruction or mental representation of a certain system, action, or operation is at stake, the necessary perception of the totality is needed.

From the initial analysis made about a certain object in its sensorial concreteness, as it is presented in reality, an abbreviated expression of that object is reached in the subject’s consciousness, that is, an abstraction is obtained, which is the condition to arrive at a theoretical appropriation of the objective world. With the formulation of this abstraction - cell that represents the essence of the whole under analysis -, the subject finds the basis for the genetic deduction of this whole through recreation. It is possible by a synthesis process of the system of relationships and connections that reflect the formation of the concept, gaining, with this, the essence of the learning object.

In this relationship between both processes, analysis and synthesis, the analysis dialectically provides the necessary abstraction to meet the general idea that organizes a whole, and the synthesis, on this basis, recreates this whole in its essential relationships and connections, providing itself the necessary abstractions for the process of recreating the concrete on theoretical basis.

The reduction process is dynamically integrated with the process of ascending from abstract to concrete in the theoretical assimilation of objective reality. Although it is a subordinate moment within this process, the reduction is, in it, a moment of fundamental importance, because it serves as a means for the realization of the whole movement from the abstract to the concrete in the formation of the concept that expresses the essence of the object of study.

Then, we have that the theoretical process that leads to the knowledge of the learning object in its essence, producing the concrete in the thought through the ascension of the abstract to the concrete, is, at the same time, by the synthesizing activity of the mind, a process of reduction from concrete to abstract. In the dialectical movement between analysis and synthesis, reduction and ascension, abstraction and generalization, the theoretical thinking activity produces the knowledge of the essence of the reality phenomena. All of these processes, which are closely related, are essential to the formation of the student’s theoretical thought that acting on the objects that make up the content of Learning Activity. With this, the students come to know the essential concrete that expresses reality in the form of concept, of knowledge of the essence of objects, and of facts and phenomena of the real world, enabling them to have a more comprehensive and in-depth understanding of everything that happens around them, thus having greater possibilities of acting in it with the capacity to transform reality.

#### The Capacity for Mental Planning

Mental planning is another element that makes up the structure of the theoretical method of action that enables the formation of the capacities of theoretical-reflective thinking through Learning Activity. In the educational process, one of the elements that enable children’s learning is related to their ability to anticipate imminent actions, to define different ways to accomplish a task, to consider the specific qualities of each variant, and to choose the best action to solve problems. This ability is defined as mental planning.

Mental actions, even the most complex and developed ones, are functions that originate in the practical and objective activity of social being, and these functions must be a means of internal activity as a transformer activity of the sensitive object world. Internal and external actions of human beings must be understood as a process that takes place in a single totality. Thus, when externalizing his mental actions and transforming objects or internalizing his external actions and modifying internal actions according to a desired objective, the individual is performing a single activity, which is essentially a dialectical activity, which can be summarized in the relationship between one mode of action and another, with the objective of reproducing the essential characteristics of the activity elements.

This way, planning is part of the theoretical action system of thought and considered as one of the main and specific functions of intellectual acts that intends to provide the means to solve problem situations. Searching for and building these systems is one of the determining factors in the process of guiding students in a given learning task, that is, mental planning is the ability to predict the results of future actions and to delimit the forms and methods of organization of the human’s own activity.

In order to understand the developing movement of mental planning, some of its characteristics must be defined: the first step is to understand that the act of planning originates from the activity. This fact happens like any other action of human beings; so, it is not different with the action of planning. Humans begin to plan their actions when they are faced with a new practical problem, and the available tools are inadequate to solve it, or must be adapted or readapted to the new conditions. The adaptation process takes place at the level and in the field of the idea, that is, at the level of an imminent action; its result is a plan of future actions. Thus, the planning action has the characteristic of being an activity-oriented action.

This specific characteristic of planning, as the basis for guiding human activity, is defined and shaped in objects. When planning their actions, the subjects recreate the images of objects and their movement in space and time, mentally producing future goals to be achieved by their actions; in other words, they create what today does not exist in the final form, but which must be carried out and executed with organized action patterns before the materialization of the act. Theoretical planning also has, as its own movement, a process that structures the hierarchy of any system of action, since it has the function of controlling the order in which any chain of operations must be carried out.

As a component of the theoretical method of action, mental planning should be treated as a special function of reflective-theoretical thinking that makes it possible to build systems of potential actions, to ideally outline a plan for future practical actions, to construct and reconstruct several variants of these actions, and the ability to correctly choose between them, considering the conditions, objectives, and other requirements of the problem proposed by the learning task.

This is a basic mental operation for the formation of theoretical thinking, since its content “consists in moving the defined (assigned by the situation) quantitative and spatial relationships of a complex object into a temporal sequence of subjective actions, into a logically complete system of actions or operations” (Magkaev, 2018, p. 306). This implies that the student constitutes a principle of organization of his acts of thought in a temporal sequence and transforms the means available for the construction of plans into objects of cognition.

This process makes it possible to master the space-time relationship of objects and phenomena of reality and, at the same time, leads to the understanding and appropriation, by the subjects, of the functional and conceptual relationships and connections. It is the development of these connections and relationships that can be defined as the birth of mental actions or thought itself, which causes a transformation in the nature of the subjects’ activity, making them take as a reference a scheme that goes from thinking of the situation and not the opposite, since, due to this development, the subjects now operate with the ability to use their own thinking to project future changes in the object of study. Action, in the process of becoming thought, fulfills the role of projecting possible object configurations, that is, mental action is able to predict the changes of an object, assuming the consequences and results that can be achieved in the future (Magkaev, 2018).

With the purpose to understand planning in its entirety, both regarding the objective basis of an action in general, and to the understanding of the method of action itself defined by the objective basis and subjective intention, it is necessary to deal with qualities such as foresight and intention. In the search for an understanding of the planning action, it is necessary to use these qualities as criteria to determine the level of its development, not only of the planning function alone, but also of its role in the totality of the theoretical method of action (Magkaev, 2018). Intentionally, during the first actions of Learning Activity, the subject of the theoretical action that was made on an object already conceived in the form of an abstraction, which contains the general relation that organizes it, makes the prediction of what are the essential relations and connections necessary to the mental planning of a scheme. This scheme allows the reduction of data from the initial abstraction to a unifying whole of those essential relations and connections of the object. This movement is essential for the process of ascending from the abstract to the concrete that characterizes the formation of theoretical thought.

The question of the origin and development of essential forms of planning is internally related to the emergence of imagination and the means of thinking symbolically. The meaning of the essential content of the mental planning action consists of a movement that goes beyond the limits of the present space and time. Action planning implies having mental control over the movement of objects and actions perceived and performed in the present, foreseeing the forms of future actions. This action essentially creates in the mind the idea of a time of action that unifies and connects the past, the present, and the future (Magkaev, 2018). This is expressed in the second action of the Learning Activity, by which the student conceives a schematic representation plan of the essential elements that constitute the totality of the object under study: the universal relation of this object, which will be reflected in the corresponding concept, is modeled under the “object, graphical, or lettered form,” constituting “the internally essential link in the process of assimilation of theoretical knowledge and generalized action procedures” (Davídov, 1988, p. 182) and thus allowing the fixation of the universal relationship of the object and its subsequent analysis toward the congregation of its multiple determinations that configure the concrete thought.

According to Magkaev (1975), in the process of developing the mental planning action there are three central aspects: the first is understanding that the planning action is always constituted by a mental search for the use of information operational systems still unknown, but possible to be carried out, and not mere memory and mental reproduction of operations already carried out; the second is the intentional and deliberate nature of the search for these systems of operations that will give rise to a mental action plan; and its logical structure and the actual construction of that structure are the particular focus of the individual’s action. And, finally, these systems are developed based on the prediction made by the subject regarding the results of future actions to a specific or determined point of qualitative deepening.

Instead of the reproduction and application of finished plans, in the action of theoretical planning, the students intentionally plan and carry out actions to know the unknown, elaborating models that are configured as efficient means of their mental activity and thus forming their capacity of theoretical analysis of reality, which reinforces the belief that Learning Activity is the most appropriate way to build capacities related to theoretical thinking.

#### The Capacity for Reflection

Reflection is the third component of the action methods that form reflective-theoretical thinking, a capacity that is fundamental for the student in Learning Activity, as it is the essence of this form of thinking that is created during the performance of this activity. “Reflection is understood as subjects’ reorientation toward their own method of action” (Magkaev, 2018, p. 307), so it is a required capacity in the entire process of carrying out the learning task, particularly in the fulfillment of evaluation and control actions that are part of the learning task. According to Davídov, the

[…] accomplishment of control and evaluation actions supposes that the students’ attention is directed to the content of their own actions, to the examination of their fundamentals, from the point of view of correspondence with the result required by the task. Such examination, by the students, of the fundamentals of their own actions, called reflection, constitutes the essential condition for these actions to be structured and to change correctly. Learning Activity and some of its components (in particular, control and evaluation) are carried out thanks to such a fundamental quality of human consciousness, as reflection is (Davídov, 1988, p. 184, our translation).

Zuckerman, 2003, p. 182–183, italics in the original) conceives reflection as a basic human capacity that, when it is highly developed, enables subjects “to consider the goals, motives, methods, and means of one’s own and other people’s actions and thoughts (the mental facet of this ability is sometimes called *metacognition*).” Another characteristic of people who have a developed reflection capacity is the possibility “to take other people’s point of view, that is, view matters from perspectives other than one’s own.” And the third element of this ability is “to understand oneself; study one’s own strong points and limitations in order to find ways to excel or to accept shortcomings,” thereby finding adequate paths to develop oneself as a human being. The introspection and the capacity that subjects have to transform themselves are fundamental elements in the development of the capacity for reflection, overcoming and transcending the limitations imposed on them in the problem situations of human existence.

Although reflection is considered a basic human capacity, its development should not be understood as natural; on the contrary, like all higher capacities of the human being, reflection is only developed through the insertion of subjects in educational processes in which this capacity is required, promoted, and constituted by their own activity in their relationship with the other subjects of this process and in contact with the most elaborate knowledge of human culture. When there is no education that provides students with an organization of activities that enable the appropriation of reflective actions of acting and thinking, this essential capacity will go on as a characteristic of few people.

Learning Activity is the main way to develop skills related to reflective-theoretical thinking of students; among these skills, reflection is carried out by the students, in an intentional and systematic way, in order to seek the discernment, the differentiation between the known and the unknown, the formulation of hypotheses about what that unknown is, and also to seek constant dialog about their own actions and those of their peers. The reflection carried out during the formation process of Learning Activity takes students to the essential investigation of the unknown and makes them request essential information about it. It also creates in them the habit of criticizing their own ideas and actions, as well as the actions and arguments of other people who are participating in the process. It gives them the arguments that are indispensable for refusing knowledge that is not based on science, reality, and truth. The development of the capacity for reflection results in the subject’s willingness to seek, evaluate, and understand the different points of view involved in understanding reality data, as well as thinking about their own actions and the actions of other people.

An outline of the capacity for reflection is already observed in the actions and thoughts of children in the final years of Kindergarten, but consolidated reflection as a general behavioral attitude is unlikely before systematic schooling. Even in the first years of elementary school, the development of reflection is only possible and really materializes if the educational process were organized at the level of Learning Activity, that is, an organization that reproduces the methods of actions that make up reflective-theoretical thinking.

In the educational process designed for elementary school children and young people, subjects can be guided to three spheres of manifestation of reflective thinking (Davídov et al., 2003, p. 71): the first one is the sphere of thought directed toward task resolution, in which the students, having in mind that reflection is “the orientation of thinking toward itself, its own processes, and products […],” become “aware of the foundations of one’s own actions” through the guiding action of their teacher. During the accomplishment of the learning task, the student is oriented toward the fulfillment of reflective actions on the theoretical way of solving it. The second sphere of reflective thinking action is communication and cooperation, which is present at the moment when, in joint action with other students, the child develops a reflection on his own actions in relation to the actions of his colleagues, in a context of “partners’ mutual understanding.” The third and last sphere of existence of reflective action concerns self-awareness. This happens when young learners exercise their reflective capacity to perceive their own limits and difficulties and establish new learning tasks for their self-development. In these tasks they select the contents and the means to solve it, bearing in mind that self-awareness “needs reflection in self-determination of its inner bearings and its means of demarcating the ‘I’ from the ‘not I’.” The development of reflective capacity through Learning Activity is, therefore, fundamental for students to develop awareness about their limitations and abilities, to understand and to transform the limits of their abilities to face new needs, and to evaluate if these needs are being met or not by the tasks performed.

It is evident, then, that one of the main functions of reflection is to reorient the subjects in relation to their own method of action. The content and meaning of the reorientation role takes place under the following conditions: the first condition is the critical assessment of the reasons for the changes in objective conditions. These objective conditions are taken as the causes of changes in their own actions, and as the review or confirmation of their worldviews, or subjective positions; the second condition refers to adaptations or adjustments in plans and intended purposes according to changes in the conditions of action; the third is the critical evaluation of the actions to verify if they still correspond to all the objective questions of a problem, that is, to its immediate results and future consequences, following each movement of the development and the logical triggering of the actions; and, finally, there is a critical examination of the full content of their method of action as a method for solving a particular set of problems (Magkaev, 2018).

When reflection is part of a problem-solving situation, it participates in the establishment of interconnected analysis and planning actions for the fulfillment of the learning task. Reflection also monitors the movement of the situation that sets in motion the transforming process of the object of study. It also controls, evaluates, and directs the movement of the subjects of activity in the given situational context. In other words, reflection is the capacity that the student uses to “achieve an effective understanding of the sense and goal of their activity” (Magkaev, 2018, p. 309).

## Conclusion

The concern on the specificities of the capacities related to theoretical thinking that are developed through Learning Activity has, in educational research, two fundamental and necessary ways of deepening. First, education must claim, as its research object, thestudy of the capacities that also constitute its scientific field of action, because to understand how the paths of the development of human capacities are established is the starting point of any educational process aimed to understand how children learn. In this sense, research in pedagogical practice must take thought capacities as its object to avoid the psychologization of processes that are psych pedagogical.

The second path understands that, in the educational process, the specificities of capacities related to theoretical thinking must be sought in the analysis of the historical movement of developing capacities. Examples of this are the capacities for reflection, analysis, and mental planning: they are the result of a movement that has its origin in the ontogenesis of child development, and, throughout this process, through the main activities, qualitative leaps occur. These leaps will form the bases for the theoretical forms of these capacities, which will be carried out through Learning Activity as a guiding activity for the development of schoolchildren. This process is only possible through the resolution of learning tasks that require the formation of these skills to solve them.

For this reason, the process of systematic resolution of learning tasks enables the constitution of the student’s theoretical thinking, as a result of the development of skills such as analysis, reflection, and mental planning, which in their dialectic relationship of unity form the conditions for the formation of reflective theoretical consciousness. The specific links of learning actions with these elements of theoretical thinking are the key to understanding the mechanisms of the influence of Learning Activity on students’ cognitive development.

Once the aspects of such an influence are understood, Learning Activity is also understood as a means for the development of capacities related to theoretical thinking and, thus, it becomes evident that theoretical thinking is an important part of education aimed at human development, which passes through the formation of its theoretical consciousness. It is the relationship between conscience and theoretical thinking, which originates in human activities, which enables subjects to transform, overcome, and create new conditions for their existence and for the environment in which they live.

Only an education that causes human development, that is, a Developmental Education, is able to enable this qualitative form of awareness, because it promotes autonomy in students for the planning, organization, execution, and evaluation of Learning Activity, leading the students to, consequently, outline creative attitudes in their relationship with the world, which is the concept of education and human being that we defend.

## Author Contributions

CC and SM made the theoretical research. SM guided the theoretical research. ÉK co-wrote and designed the article. SM reviewed the manuscript. All authors contributed to the article and approved the submitted version.

## Conflict of Interest

The authors declare that the research was conducted in the absence of any commercial or financial relationships that could be construed as a potential conflict of interest.
